# Teachers’ collective efficacy with regard to inclusive practices—characteristics of a new scale and analyses from Canada, Germany and Switzerland

**DOI:** 10.3389/fpsyg.2025.1530689

**Published:** 2025-06-13

**Authors:** Margarita Knickenberg, Harry Kullmann, Sergej Wüthrich, Caroline Sahli Lozano, Tim Loreman, Umesh Sharma, Elias Avramidis, Pearl Subban, Stuart Woodcock

**Affiliations:** ^1^Faculty of Arts and Humanities, Paderborn University, Paderborn, Germany; ^2^Institute for Research, Development and Evaluation, University of Teacher Education, Bern, Switzerland; ^3^Faculty of Education, Concordia University of Edmonton, Edmonton, AB, Canada; ^4^University of Nottingham Ningbo China, Ningbo, China; ^5^Department of Special Education, University of Thessaly, Volos, Greece; ^6^School of Education and Professional Studies, Griffith University, Brisbane, QLD, Australia

**Keywords:** collective efficacy of teachers, inclusive instruction, collaboration, managing behavior, scale validation, Canada, Germany, Switzerland

## Abstract

**Introduction:**

While teachers’ individual and collective efficacy has been extensively studied with regard to promoting students’ academic success, teachers’ collective efficacy regarding inclusive practices has been largely neglected thus far, especially from an international perspective. International comparisons are of particular interest to any country or school system, respectively, as they can help to identify alternative approaches and opportunities for inclusive school development. The scale examined in this paper is ascertaining teachers’ collective efficacy with regard to inclusive education (TEIP-C) and is derived from a scale measuring (individual) Teachers’ Efficacy for Inclusive Practices (TEIP). This scale comprises three subscales termed *Inclusive Instruction*, *Managing Behavior* and *Collaboration*. Our major aim was to validate the tripartite structure of the original TEIP scale for the new TEIP-C scale and to demonstrate measurement invariance of the latter employing an international sample.

**Methods:**

The sample comprised 897 teachers from Canada, Germany and Switzerland. Different Confirmatory Factor Analysis (CFA) models were combined with Exploratory Structural Equation Models (ESEM). Measurement invariance across countries was examined by means of a multiple group confirmatory factor analysis (MGCFA) approach. Afterwards, the variables gender, age and teaching experience were included simultaneously as predictors of collective teaching efficacy to specify a multiple indicator multiple cause model (MIMIC).

**Results:**

We successfully validated the tripartite structure of the original TEIP scale for the new TEIP-C scale and demonstrated its measurement invariance employing samples from Canada, Germany, and Switzerland. Based on similar validations, it now appears possible for researchers to freely combine either of the six subscales focusing on teachers’ individual or collective efficacy with regard to inclusive education in their questionnaires in future studies. While the three country samples did not differ regarding Inclusive Instructions, significant differences in favor of Canadian teachers became apparent for Collaborations (compared to both, Switzerland and Germany) as well as Managing Behavior (Germany).

**Discussion:**

Overall, the results underline the comparably high standards of inclusive teaching in Canada. Additional differences on the basis of the two subscales just mentioned pointed to somewhat lower ratings of collective teacher efficacy with respect to inclusive education by female teachers in Canada and Germany and older teachers in Switzerland.

## 1 Introduction

As more schools around the globe become inclusive, the student body in many classes is getting more diverse regarding various dimensions. In order to make the shift to inclusive education a success, a variety of conditions summarized in “the 4 As” of Availability, Accessibility, Acceptability, and Adaptability have to be in place (UN-CRPD, 2016). To meet the heterogeneous needs of all learners, teachers are crucial, both in terms of their individual competences and their collective performance as part of a multi-professional team (e.g., Paulsrud and Nilholm, 2020; Pozas and Letzel-Alt, 2023; Subban et al., 2023). Searching for factors of success, many studies emphasize teachers’ attitudes towards inclusion, their self-efficacy as well as their collective efficacy as being essential (e.g., Subban et al., 2023; Wray et al., 2022; Zee and Koomen, 2016).

Collective efficacy is rooted in social cognitive theory (Bandura, 1997) and can be defined as “a group’s shared belief in its conjoint capability to organize and execute the courses of action required to produce given levels of attainment” (Bandura, 1997, p. 477). Individualized teaching adapted to students’ diversity requires well-functioning teams of teachers characterized by high solidarity, mutual appreciation and respect, and shared responsibility which are closely connected to their collective efficacy (e.g., Parker et al., 2006). Accordingly, school teams achieving a high degree of collective efficacy appear to set themselves higher goals and to pursue them with an elevated persistence. Such goals, according to Goddard et al. (2000), can be regarded as normative expectations for the individual teacher, influencing their beliefs about teaching and learning as well as their performance in the classroom. Other putative mechanisms in this respect comprise peer learning, peer-induced professional development and different yet opaque routes of spillover effects due to highly effective colleagues (e.g., Jackson and Bruegmann, 2009). In the present study, teachers’ collective efficacy for inclusive practices refers to the shared belief among teaching staff that they, as a group, are capable of successfully implementing inclusive teaching strategies, managing heterogeneous classrooms, and collaborating with parents and other professionals to support all learners.

It is important to note that teachers’ individual self-efficacy is supposedly highly context specific, i.e., they “feel efficacious for teaching particular subjects to certain students in specific settings, and they can be expected to feel more or less efficacious under different circumstances” (Goddard et al., 2000, p. 482). Therefore, considering the collective approach necessary for successful education in inclusive settings as the “circumstance” of interest, especially targeted measures of collective efficacy of teachers in inclusive settings have to be employed.

Collective beliefs and individual self-efficacy are interconnected (Skaalvik and Skaalvik, 2007). Teachers’ collective efficacy is known for being linked to students’ achievements (e.g., Tschannen-Moran and Barr, 2004; Zee and Koomen, 2016). Bandura interestingly proposed in this respect that “the totality of teachers’ beliefs in their own efficacy is just as predictive of school performance as the totality of teachers’ beliefs in their schools’ efficacy as a whole” (Bandura, 1997, p. 481).

## 2 Measuring teachers’ collective efficacy in inclusive education internationally

While teachers’ individual as well as collective efficacy have been extensively studied with regard to promoting students’ academic success, teachers’ collective efficacy with respect to inclusive practices has been largely neglected thus far, especially from an international perspective (e.g., Sharma et al., 2024). One of the approaches on a national base is the adaption of the *Collective Teacher Belief Scale* (Tschannen-Moran and Barr, 2004, item example: How much can teachers in your school do to produce meaningful student learning?) for students with intellectual difficulties (ID, Wilson et al., 2020, item example: How much can teachers in your school do to produce meaningful student learning for a child with ID?’). This scale, apparently, is targeted towards the teaching of a rather small fraction of students to care for in inclusive settings. Additionally, in the respective study, the two-factor structure originally presented by Tschannen-Moran and Barr (2004) could not be reproduced and no data on confirmatory factor analysis were provided (Wilson et al., 2020).

The scarcity of questionnaire scales that have successfully demonstrated international validity using criteria such as measurement invariance may be one reason for the paucity of research in this area. International comparisons are of particular interest for each country or school system, respectively, as they can help to identify alternative approaches and possibilities for inclusive school development.

### 2.1 From individual to collective teacher efficacy for inclusive practices (TEIP)

The need for an instrument to measure teachers’ collective self-efficacy in inclusive educational contexts was addressed by Subban et al. (2023) following the earlier establishment and characterization of a scale ascertaining (individual) Teachers’ Efficacy for Inclusive Practices (TEIP, Sharma et al., 2012). The scale was initially cooperatively developed by an international research group and comprised 18 items. They were examined in a sample consisting of *N* = 607 pre-service teachers from Australia, Canada, Hongkong and India (Sharma et al., 2012). A factor analysis revealed three factors with six items each: The factor *Efficacy to use Inclusive Instructions* relate to strategies that promote the inclusion of all learners (e.g., “I can provide appropriate challenges for very capable students.”). Items that affect individual’s perceptions of teacher efficacy in working with parents and other professionals were aggregated to the factor *Efficacy in Collaboration* (e.g., “I can assist families in helping their children do well in school.”). The third factor termed *Efficacy in Managing Behavior* covers items referring to teachers’ efficacy in dealing with disruptive behavior (e.g., “I am confident in my ability to prevent disruptive behavior in the classroom before it occurs.”). The internal consistency of the three scales ranged between *α* = 0.85 and α = 0.93, indicating a reliable measure of teachers’ individual efficacy beliefs to teach in inclusive classrooms (aka TEIP).

In a recent subsequent study, the TEIP scale was adapted for the measurement of teachers’ collective efficacy to implement inclusive practices (TEIP-C; Sharma et al., 2024). The TEIP-C scale captures this construct by assessing teachers’ perceptions of their colleagues’ collective capacity to create inclusive learning environments through two core domains: inclusive pedagogical practices and proactive engagement. This conceptualization builds directly on Bandura’s (1997) notion of collective efficacy (see above), yet specifically adapts it to the demands of inclusive education. In line with TEIP, TEIP-C consists of 18 items affecting teachers’ perceptions about the capacity of their colleagues to influence the inclusion of all learners (see Sharma et al., 2024 for a documentation of the entire scale). *N* = 1,523 teachers from Canada, Greece, Italy and Switzerland participated in the survey. Results of a Principal Component Analysis (PCA) suggest a two-factor solution representing *Engagement* (e.g., “Teachers in my cohort prevent disruptive behavior”) and *Inclusive Pedagogies* (e.g., “Teachers in my cohort use a variety of assessment strategies in order to determine if all children in a class are learning”). The total TEIP-C-Scale as well as both subscales displayed strong reliability indicated by Cronbach’s Alpha (0.95, 0.93, and 0.91, respectively, Sharma et al., 2024).

### 2.2 Perspectives on inclusive education in Canada, Germany and Switzerland

The study presented here builds on the prior research just described, focusing on Canada (CAN) and Switzerland (SUI) and additionally including the various perspectives of teachers from Germany (GER). While Canada and Germany both ratified the UN-Convention on the Rights of Persons with Disabilities (UN-CRPD) in 2010 and 2009, respectively, Switzerland acceded in 2014. As Switzerland refused from signing the optional protocol allowing individual actions and claims, the Swiss Federal Council refrains from interpreting disability laws to include hard obligations with regard to including children with disabilities into mainstream classes (Sharma et al., 2024).

However, while the UN-CRPD is a binding law only in Canada and Germany, its goals are shared on a national level in all three countries for at least a decade. All countries are also similar in that they are a federation in which the responsibility for decisions regarding the educational system, e.g., the amount and distribution of resources or the set-up of teacher education, mainly lies on the level of the respective unit, i.e., Provinces, Lander or Cantons (e.g., Sokal and Katz, 2015). The resulting differences between the various federal states comprise all relevant aspects regarding inclusive education, making it hard in each country to sum up valid evaluation data on a national level (e.g., Loreman, 2014). According to Miesera et al. (2021), the consistency with regard to inclusion-related teacher education is higher in Germany in comparison to Canada. However, as Wray et al. (2022) point out, there has been a longer history of system expectations with regard to inclusive education in Canada. Germany, for example, is keeping up with a highly stratified secondary school system as well as with an elaborated system of special schools. Although this is true for most though not for all Lander, Germany is seen as “still adapting to better align with global agreements” (Wray et al., 2022. p.5).

Additionally, and again for all of the three countries, some responsibilities regarding the school system or the situation of individual schools lie in the hand of regional government authorities such as school districts. Cultures, policies, and practices – as major dimensions of the well-known self-evaluation tool *Index of Inclusion* (Booth and Ainscow, 2016) – may therefore well vary to a great extent between individual schools in each country (Subban et al., 2023). The Canadian school system is of particular interest, especially from a European perspective, since it has long been seen as a best practice example with respect to inclusive teaching although this has also been met with some skepticism (e.g., Sokal and Katz, 2015; Merz-Atalik, 2022). In empirical studies, teachers from Canada regularly differ positively with regard to inclusion-related attitudes, intentions, or (individual) self-efficacy in comparison with their counterparts from other countries, including Germany or Switzerland (Miesera et al., 2021; Sharma et al., 2006).

In prior studies originating from the same international project as this paper but focusing on different scales, teachers from Canada rated (significantly) higher than their German and Swiss counterparts with regard to various aspects of inclusive teaching, including *Attitudes towards Inclusive Education* (AIS, Sharma and Jacobs, 2016), (individual) *Teacher Efficacy for Inclusive Practices* (TEIP, Sahli Lozano et al., 2023, 2024; Sharma et al., 2012), and Teachers’ *Intention to Teach in Inclusive Classrooms* (ITICS, Sharma and Jacobs, 2016; Sharma et al., 2024). The absolute differences between Germany and Switzerland appeared to be mostly small excluding ITICS with prominently higher values for German teachers (Cohens’ *d*: 1.08, c.f. Sahli Lozano et al., 2024). When predicting ITICS on the basis of two AIS-Subscales (*Beliefs Regarding Inclusive Education* and *Feelings Regarding Inclusive Education*) and three TEIP-Subscales (*Efficacy in Inclusive Instructions*, *Efficacy in Managing Behavior*, and *Efficacy in Collaboration*), the latter subscale appeared to significantly predict ITICS for Canadian and German teachers, with *Managing Behavior* as well as *AIS-Feelings* being predictive with regard to the Swiss sample and *TEIP-Instructions* for the German teachers (Sahli Lozano et al., 2024).

The paper examining the TEIP-C scale mentioned above compared various perspectives of teachers regarding inclusive education, including data from Canada and Switzerland (Sharma et al., 2024). With regard to TEIP-C, analyses were limited to an ANOVA-based comparison between the samples utilizing all 18 Items on a six-point Likert-scale (scale minimum/maximum: 18/108). The total mean values for Canada (CAN) were the highest (arithmetic mean (*M*) CAN: 81.37, standard deviation (*SD*): 15.44) with Switzerland (SUI) not lagging behind significantly (*M*: 80.24, SD: 10.32, Sharma et al., 2024).

### 2.3 Research objectives

Against the background described above, the paper presented here co-examines the newly developed scale assessing teachers’ collective efficacy with regard to inclusive practices (TEIP-C, Sharma et al., 2024) in a global context, focusing on teachers’ assessments from Canada, Germany, and Switzerland. The first purpose of this study is to characterize the TEIP-C scale by evaluating its dimensionality and factorial structure. Since in prior analyses TEIP was shown to comprise three sub-scales (Sharma et al., 2012) while TEIP-C displayed a two-factorial structure (Sharma et al., 2024) to compare a 2- with a 3-factor solution is among the objectives. Another aim is to test for measurement invariance across the three respective states as a basis of international validity and to perform selected comparisons regarding the relevance of individual background variables such as gender and teaching experience.

## 3 Methodology

### 3.1 Sample and procedure

The analysis is integral part of an international research project aiming at predicting teachers’ intentions to teach in inclusive classrooms in a global context (Sahli Lozano et al., 2024; Subban et al., 2023; Subban et al., 2024). Within the scope of this project, questionnaires were administered online to 897 preschool, primary and secondary school teachers (77.3% female) in the Canadian provinces of Alberta and British Colombia (*n* = 281), the German Bundesland of North Rhine-Westphalia (*n* = 257) and the German-speaking part of Switzerland (*n* = 359). Male and female teachers are equally represented in the three datasets (female in %: CAN: 75.3; GER: 75.3; SUI: 77.8).

Approximately half of the teachers in the total sample reported having a teaching experience of 10 years or more (52.3%), while beginners (1–4 years: 26.5%) and teachers with 6 to 10 years of experience (21.2%) make up for one forth or one fifth of the sample, respectively. All of the following age groups were fairly equally represented: under 30 years: 22%; 30–39 years: 27.40%; 40–49 years: 23.8%; over 50 years: 26.8%. Data were collected between June and November 2021 in Canada (CAN), February and June 2022 in Germany (GER), and June 2019 and December 2022 in Switzerland (SUI).

### 3.2 Instrument

The collective version of *Teachers’ Efficacy for Inclusive Practices* Scale (TEIP-C) was measured using 18 items concerning teachers’ “capacity of their peers to influence the success of routine classroom activities in creating an inclusive classroom environment” (Sharma et al., 2024, p. 8). Within the scope of this study, teachers were asked to rate their agreement on the various items on a 6-point Likert scale ranging from 1 (=strongly disagree) to 6 (=strongly agree).

### 3.3 Data analyses

All analyses were performed in M*plus* Version 8.9 (Muthén and Muthén, 1998–2023). Estimations were done using robust maximum-likelihood estimator (MLR) which accounts for the data’s non-normal distribution referring to robust standard errors. In order to evaluate the dimensional structure of TEIP-C, a confirmatory and exploratory structural equation modelling framework was used (Asparouhov and Muthén, 2009). Confirmatory factor analyses (CFA) can be characterized, among other features, by the fact that the relationship between item and latent factor have already been defined *a priori* and that cross-loadings are not allowed, so that each manifest item loads only on one single latent factor.

Constraining cross loadings to be zero might result in inflated CFA factor correlations and biased estimates (Marsh et al., 2009). Therefore, CFAs often are rejected due to poor model fit indices and are considered as being too restrictive (Marsh et al., 2010; Marsh et al., 2011; van Zyl and ten Klooster, 2022). In contrast to CFAs, exploratory structural equation modelling (ESEM) frameworks consider that items often show small residual associations with other factors besides the target factor (Asparouhov and Muthén, 2009). Even though the latent factor structure is also predefined in ESEMs, cross loadings are allowed (usually these should be close to zero, but not forced to be zero).

**Figure 1:**
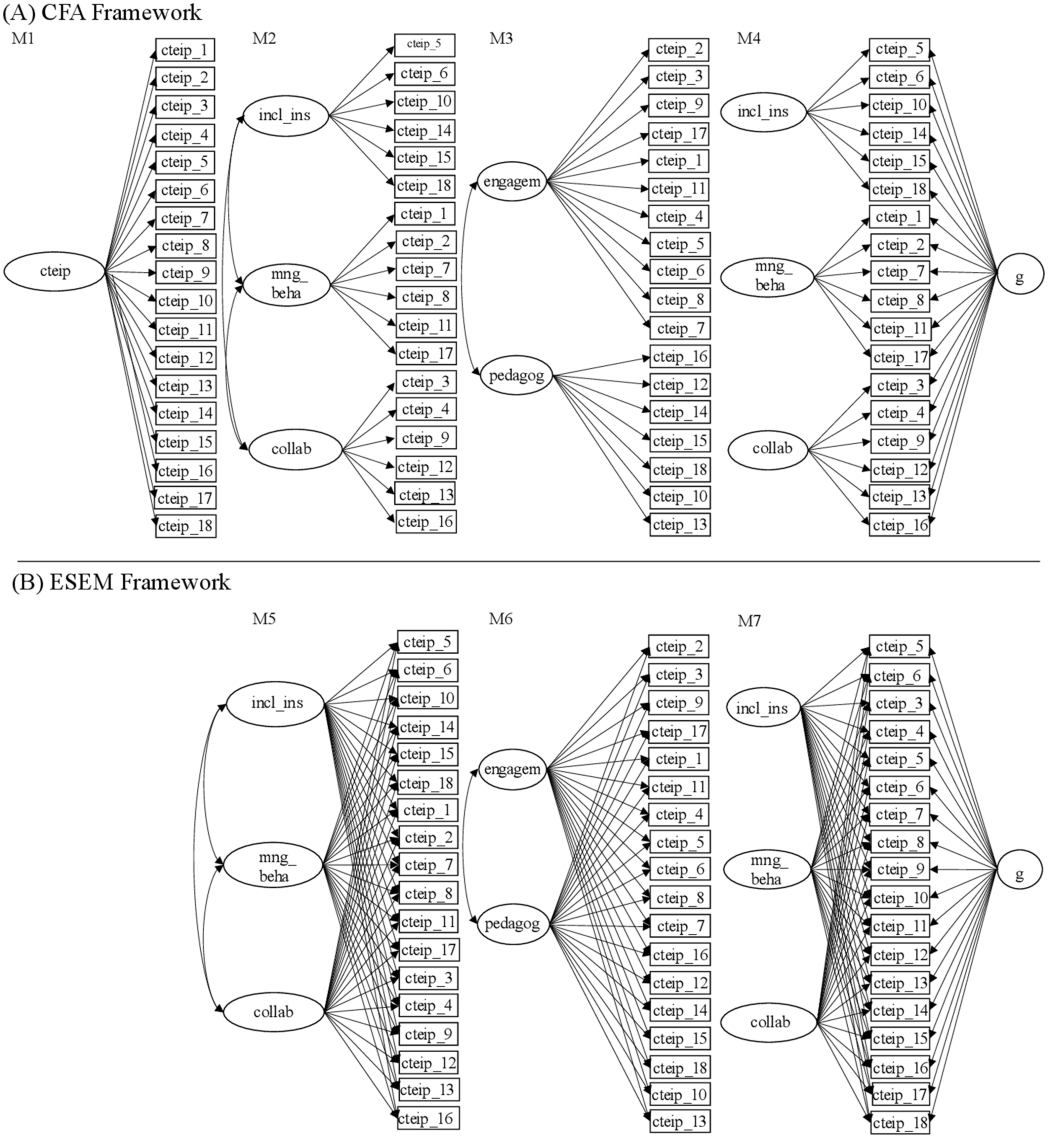
Graphical representations of the tested models’ theoretical structure (M1 to M7). cteip: Collective teachers’ efficacy for inclusive practices. incl_ins: Efficacy to use inclusive instructions. mng_beha: Efficacy in managing behavior. collab: Efficacy in collaboration. engagem: Efficacy in proactive engagement. pedagog: Efficacy in pedagogical engagement. g: General factor. **(A)** Describes the CFA framework, **(B)** Describes the ESEM framework.

Thus, in addition to different theory-driven CFA models, we also performed a series of ESEM on the TEIP-C data. In detail, the following models were tested (see Figure 1): (M1) CFA with one general latent factor, (M2) CFA with three first-order correlated latent factors (analogously to TEIP; Sharma et al., 2012), (M3) CFA with two first-order correlated latent factors (in line with the TEIP-C structure proposed on the basis of PCA in Sharma et al., 2024), (M4) bifactorial CFA with three uncorrelated latent factors and an additional general factor, (M5) ESEM with three first-order latent factors, (M6) ESEM with two first-order latent factors and finally, (M7) bifactorial ESEM with three uncorrelated factors and an additional general factor.

In ESEM frameworks, target rotation was used for first-order models to allow for cross-loadings, in bifactor models, orthogonal rotation was applied to take the independent relationship between the general factor and the specific ones into account (van Zyl and ten Klooster, 2022).

To evaluate the models’ fit to the empirical data, the following, sample-size independent model fit indices were considered: Comparative Fit Index (CFI), Tucker-Lewis Index (TLI), Standardized Root Mean Square Residual (SRMR), and Root Mean Square Error of Approximation (RMSEA). CFI and TLI > 0.90 indicate an appropriate data fit to the theoretically assumed model, whereas SRMR<0.08 and RMSEA<0.06 are considered as acceptable (Hu and Bentler, 1999).

Chi-square (χ^2^) and its associated degrees of freedom (*df*) were also reported, although they are known for being sensitive to sample size, data’s non-normality and model complexity (Yuan and Bentler, 2004). Additionally, the respective difference test has been criticized as being too restrictive (Meade et al., 2008). Next to the model fit indices, parameter estimations like factor loadings and factor correlations were also evaluated to check for model fit. In addition, models with the lowest AIC (Akaike Information Criterion), BIC, and aBIC ([adjusted] Bayesian Information Criterion) should be favored. Since CFA models are nested in ESEM models (CFAs have more restrictions), chi-square difference tests or differences in RMSEA and CFI were also considered. A significant chi-square difference as well as RMSEA- and CFI-differences >0.015 and >0.01, respectively, are indicative of worse fit in the more restrictive model, suggesting the less restrictive model should be preferred (Chen, 2007). Based on these criteria, the best fitting model among the competing CFA and ESEM models described above was chosen for further analyses.

Subsequently, measurement invariance across countries was examined by means of a multiple group confirmatory factor analysis approach (MGCFA; Jöreskog, 1971). Only if the TEIP-C scale or its subscales are invariant across groups, the scale can be presumed to measure the same trait in the different groups and meaningful conclusions from comparisons between groups can be drawn (Brown, 2015). Thus, establishing configural, metric, and scalar invariance is not only a statistical requirement but also a necessary condition for valid cross-national interpretation of latent mean differences in collective teacher efficacy for inclusive practices. In this context, it is important to note that the subsequent comparisons between countries are based on *latent mean differences*. Latent means refer to the average values of unobserved (latent) variables, which are estimated based on the underlying measurement model rather than raw item responses (Little et al., 2007). Such comparisons are considered more valid than observed mean comparisons because they are less affected by measurement error and group-specific response tendencies (Brown, 2015). The different levels of invariance were checked step by step – from the least restrictive model (configural measurement invariance) to the more restrictive model (scalar measurement invariance) – by adding restrictions successively.

If configural invariance is given, the number of manifest indicators and their loading patterns do not differ significantly from each other across the countries. Metric invariance is examined by restricting the factor loadings to be equal in the different groups. In detail, it is tested if the factor loadings are equivalent between groups and if the measurements of the latent factors refer to the same metric of items. Scalar invariance is established if also the intercepts are equal across the countries.

Since the different models are specified by adding restrictions, they are hierarchically structured and therefore nested. If the added constraints do not lead to significant decline in model fit, the more restrictive model will be preferred, and measurement invariance is proven. The (Satorra-Bentler corrected) Chi-square difference test was used to evaluate the competing models (Hu and Bentler, 1999). In addition, the CFI-difference (ΔCFI≤0.01; Chen, 2007) was also considered giving the disadvantages of the Chi-square difference test. For the final model, McDonald’s *ω* was estimated to evaluate the subscales’ reliability both for the total sample and for Canada, Germany and Switzerland separately. According to Kline (2023), values ≥0.70 indicate a good reliability.

At last, the variable gender (male vs. female) as well as the variables age and teaching experience were included simultaneously as predictors of collective teaching efficacy to specify a multiple indicator multiple causes model (MIMIC; Marsh et al., 2013). A MIMIC model resembles a multivariate regression model in which latent variables are regressed on predictors.

## 4 Results

### 4.1 Factorial structure of TEIP-C

To examine the dimensionality of TEIP-C, different CFAs and ESEMs were tested for the total sample. Detailed information on the model fit indices are presented in Table 1. Results of an unidimensional CFA (M1; *χ*^2^ (135) = 1,307.72, RMSEA = 0.102; CFI = 0.82; SRMR = 0.06) revealed unsatisfactory model fit indices. A correlated two first-order factor structure seems to be inappropriate for the present sample which is reflected in a poor model fit (M3; *χ*^2^ (134) = 1,049.44, RMSEA = 0.090; CFI = 0.86; SRMR = 0.06). While a bifactorial CFA with three uncorrelated factors and an additional general factor (M4; *χ*^2^ (117) = 393.90, RMSEA = 0.053; CFI = 0.96; SRMR = 0.03) showed acceptable model fit indices, the loadings on the target factors are weak (e.g., for the factor *Inclusive Instructions*: −0.003 ≤ *λ* ≤ 0.445).

**Table 1:** Model fit indices.

Model	Type	Number of parameters	χ^2^	*df*	CFI	TLI	RMSEA	90% CI	SRMR	AIC	BIC	(a)BIC
CFA framework												
M1	unidimensional model	54	1,307.72	135	0.82	0.80	0.102	[0.097, 0.107]	0.06	33,888.60	34,144.33	33,972.84
M2	three first-order factor model	57	657.38	132	0.92	0.91	0.069	[0.064, 0.074]	0.05	32,852.03	33,121.97	32,940.95
M3	two first-order factor model	55	1,049.44	134	0.86	0.84	0.090	[0.085, 0.095]	0.06	33,462.17	33,722.63	33,547.97
M4	bifactor model	72	393.90	117	0.96	0.95	0.053	[0.047, 0.059]	0.03	32,456.85	32,797.83	32,569.18
ESEM framework												
M5	three first-order factor model	87	430.17	102	0.95	0.93	0.062	[0.056, 0.068]	0.03	32,503.35	32,915.36	32,639.08
M6	two first-order factor model	71	586.90	118	0.93	0.91	0.069	[0.063, 0.074]	0.04	32,758.30	33,094.54	32,869.06
M7	bifactor model	102	208.70	87	0.97	0.95	0.051	[0.045, 0.058]	0.02	32,319.41	32,802.46	32,478.54
M8	ESEM (M5) within CFA	87	429.97	102	0.95	0.93	0.062	[0.056, 0.068]	0.03	32,503.35	32,915.36	32,639.08
Measurement invariance												
M5.1	configural model	261	777.50	306	0.94	0.90	0.074	[0.068, 0.081]	0.03	32,039.51	33,275.55	32,446.69
M5.2	metric model	171	860.40	396	0.94	0.93	0.065	[0.059, 0.071]	0.06	32,107.48	32,917.30	32,374.26
M5.3	scalar model	141	987.72	426	0.93	0.92	0.069	[0.063, 0.074]	0.06	32,194.15	32,861.90	32,414.13

*df*, degrees of freedom; CFI, Comparative Fit Index; TLI, Tucker-Lewis Index; RMSEA, Root Mean square Error of Approximation with 90% confidence interval (CI); SRMR, Standardized Root Mean Square Residual; AIC, Akaike Information Criterion; (a)BIC, (adjusted) Bayesian Information Criterion.

When comparing a three first-order factor CFA (M2; *χ*^2^ (132) = 657.38, RMSEA = 0.069; CFI = 0.92; SRMR = 0.05) to a corresponding ESEM, the latter appears to fit the data slightly better (M5; *χ*^2^ (102) = 430.17, RMSEA = 0.062; CFI = 0.95; SRMR = 0.03). Also, the CFI-difference (ΔCFI = 0.03) and the (Satorra-Bentler corrected) Chi-square difference test (Δ*χ*^2^ = 206.32, Δ*df* = 30, *p* ≤ 0.001) supports the choice on the less restrictive ESEM model. In terms of the model fit indices, the two first-order factor ESEM (M6) performs slightly worse compared to the three factorial ESEM (M5). Additionally, the crossloadings on the target factor are relatively high (e.g., crossloadings for the target factor *Engagement* 0.419 ≤ λ ≤ 0.571) which should be avoided in ESEM contexts. The AIC, BIC and aBIC values also indicate a preference for the three-factor over the two-factor ESEM model.

The bifactorial ESEM solution with three uncorrelated factors and an additional general factor (M7) fits the data quite well in terms of model fit. However, only the loadings on the general factor are sufficiently high (0.520 ≤ λ ≤ 0.797). The factor loadings on the target factors are partly weak and/or not significant (e.g., for the factor *Inclusive Instructions*: −0.109 ≤ λ ≤ 0.381). As stated above with regard to M1, a single general factor does not fit the data well.

At this point, it can be stated that the three first-order factor ESEM model (M5) represents the data from Canada, Germany and Switzerland best based on model fit indices and parameter estimations. The three theoretically assumed factors *Inclusive Instruction*, *Managing Behavior* and *Collaboration* are considered as confirmed. The respective model (M5) was used for subsequent analyses.

To check the factorial structure of the final ESEM model for stability when transferred into a CFA model, an “ESEM within CFA” (M8) analysis was performed. In concrete terms, this means that the (unstandardized) factor loadings (both the target loadings and the crossloadings) of the ESEM model were used as starting values of an ordinary CFA to enable further analyses. The transfer of an ESEM solution into a CFA model is successful if the model fit indices can be reproduced (van Zyl and ten Klooster, 2022). In this case, the values of M5 and M8 correspond to each other (Table 1).

### 4.2 Measurement invariance across countries

In a next step, measurement invariance across Canada, Germany and Switzerland was examined using the three first-order ESEM (M5 in Figure 1) as base model. As presented in Table 1, the configural model shows moderate model fit (M5.1). The restriction of the factor loadings in the course of the metric model examination (M5.2) results in a significant Chi-square difference test (M5.2 vs. M5.1; Δ*χ*^2^ = 125.74, Δ*df* = 90, *p* = 0.01). Nonetheless, under consideration of the CFI-difference (ΔCFI<0.01) metric invariance is given.

To examine scalar invariance (M5.3), the intercepts were restricted to be equal across the countries. This fixation also leads to a significant Chi-square difference (M5.3 vs. M5.2; Δ*χ*^2^ = 154.01, Δ*df* = 30, *p ≤ 0*.001). However, if the CFI-difference is considered again for the model evaluation (ΔCFI≤0.01), it can be assumed that scalar measurement invariance is given. In concrete terms, additional restrictions in the model do not lead to a remarkable decline in model fit, and thus, the more restrictive model can be preferred.

To subsume, the three first-order factor ESEM model withstands the examination of configural, metric and scalar measurement invariance and hence, it is statistically sound to compare latent means and associations between the three countries on collective teacher efficacy and therefore, it meets the statistical requirement for cross-national comparisons.

### 4.3 Factor reliability and factor correlations

The internal consistencies of the three empirically confirmed factors are satisfactory for the overall sample (0.88 ≤ *ω* ≤ 0.91; Table 2). Also, the McDonald’s ω values for the individual countries are above the threshold of 0.70, so that reliable factors can be assumed.

**Table 2:** Reliabilities, correlations and latent means.

Factor	(1)	(2)	(3)	*M*_scale_ (*SD*)	*M*_CAN_ (*SD*)	*M*_GER_ (*SD*)	*M*_SUI_ (*SD*)
(1) Inclusive Instruction		0.72***	0.77***	4.52 (0.85)	0	−0.09 (0.12)	−0.12 (0.11)
(2) Managing Behavior			0.66***	4.33 (0.85)	0	−0.22** (0.08)	0.08 (0.08)
(3) Collaboration				4.36 (0.87)	0	−0.30* (0.12)	−0.42** (0.14)
ω	0.91	0.91	0.88				
ω_**CAN**_	0.94	0.94	0.91				
ω_**GER**_	0.88	0.90	0.87				
ω _**SUI**_	0.90	0.90	0.84				

Bold type: correlations; Mfactor, absolute scale means; SD, standard deviation; MCDN, latent means in Canadian sample (fixed to be zero); MG, standardized latent mean in German sample (deviation from Canadian mean); MCH, standardized latent mean in Swiss sample (deviation from Canadian mean); *p ≤ 0.05; **p ≤ 0.01; ***p ≤ 0.001.

The three factors correlate moderately with each other (0.66 ≤ *r* ≤ 0.77; *p ≤ 0*.001). When comparing the factors’ correlations of the three factor first-order ESEM model with those from the corresponding CFA model, it becomes apparent that the last-mentioned values are more pronounced (0.77 ≤ *r* ≤ 0.91; *p ≤ 0*.001). According to van Zyl and ten Klooster (2022), the model with the smallest factor correlations should be preferred as this correlation represents “the level of unique distinction between factors” (p. 10). This again confirms the preference for the three factor first-order ESEM model.

### 4.4 Latent mean differences across countries

On the basis of latent means and by taking Canada as reference group (with its latent means constrained to zero) significant differences are found for Germany regarding *Managing Behavior* (*M* = -0.22; *p = 0*.010) as well as *Collaboration* (*M* = -0.30; *p = 0*.016) and for Switzerland regarding *Collaboration* (*M* = -0.42; *p = 0*.003). Apparently, the Canadian teachers in our sample show higher levels of collective efficacy with regard to teaching in diversity-enriched classes to at least some extent compared to the two central European countries.

### 4.5 ESEM MIMIC

Based on a subsequently employed ESEM MIMIC, the factor gender (1 = male, 2 = female) appears to have a small effect in the Canadian sample on *Managing Behavior* (*β* = 0.16, *p* = 0.008) and *Collaboration* (*β* = 0.16, *p* = 0.009) and also in the German sample *Managing Behavior*: *β* = 0.14, *p* = 0.046; *Collaboration*: *β* = 0.17, *p* = 0.018. Thus, female teachers tend to have higher values for two out of three dimensions of collective efficacy both in Canada and in Germany. There is no gender effect found on collective efficacy in the Swiss sample.

Age-related effects were only found in the Swiss sample regarding *Inclusive Instructions* (*β* = −0.20, *p* = 0.008) and *Managing Behavior* (*β* = −0.18, *p* = 0.021). This indicates that older teachers in Switzerland are less pronounced in their collective efficacy to at least some extent. Teaching experience has no significant effect on teachers’ collective efficacy in any of the three countries.

## 5 Discussion

The aim of this study was to examine the factorial structure of the TEIP-C scale and to report selected cross-national comparisons for Canada, Germany and Switzerland regarding the instrument’s factors. Employing a CFA- and ESEM-framework, a three first-order factor ESEM model met the requirements best. The respective model withstands the examination of measurement invariance and each factor displays satisfying reliability with regard to the current sample.

Latent mean differences as an approach to country comparisons indicate for Canadian teachers to hold a somewhat higher collective efficacy in comparison with their German and Swiss colleagues. This finding differs from earlier analyses described above where Swiss teachers did not differ from their Canadian counterparts on the basis of the 18-item-total score of TEIP-C and when comparing the manifest mean scores. The current study, by comparing three latent factors of which scalar invariance is successfully proved, is able to elucidate significant differences in collaboration-related self-efficacy between the Canadian and the Swiss Teachers in the sample. Since significant differences are found between Canadian and German teachers for two out of three factors, the teacher ratings undermine the notion of a more successful inclusive education in the Canadian provinces Alberta and BC because good and successful practice is known to be the best predictor for high and positive levels of self-efficacy (e.g., Tschannen-Moran and McMaster, 2009), at least on the individual level.

While female teachers report higher self-efficacy in Canada and Germany for two of the three subscales tested, gender does not display an effect with regard to teachers’ collective efficacy in Switzerland. It is interesting to note in this context that the gender ratio is similar across all three country samples. It would be interesting to link the respective data to observations regarding the underlying inclusive educational practices, i.e., *Managing Behavior* in classrooms and *Collaboration* with colleagues and parents in order to develop hypotheses about why in Switzerland but not in the two other countries male and female teachers show equivalent levels of collective self-efficacy in this respect.

It is tempting to speculate how different levels of teachers’ self-efficacy might originate except for a rather global notion of “reciprocal causality” (Goddard et al., 2000, p. 483) also outlined above, according to which the individual performance and efficacy fuels the collective efficacy as well as the group performance and vice versa. In other words: “Teachers are thus producer and products of microenvironments within a larger school milieu” (Bandura, 1997, p. 249).

One of the underlying processes responsible for the level of individual as well as collective teacher efficacy may of course be the collaboration among teachers. While Ronfeldt et al. (2015) propose an “individualistic” as well as “collective” mechanism by which teams of teachers supports student achievement, it seems worthwhile to exchange the outcome measure student achievement for the level of self-efficacy gained in a group of teachers. Both routes therefore inspire the theory on how teachers gain a certain level of individual as well as collective sense of efficacy in a given school. Accordingly, one could think of teachers gaining above average levels of self-efficacy by rather personal routes of professional development and reflexions, experiences and successes while they collaborate with others. These individuals would be able to acquire individual gains from their collaboration independently of whether or not the respective team of teachers is collaborating and teaching and having a group-related self-efficacy above average or not. Ronfeldt et al. (2015) propose this mechanism because they find teachers with above average success in teaching independently from the level of collaboration among their colleagues at school.

The collectivist mechanism on the other hand might explain for comparably high levels of teacher self-efficacy that is – at least in part – independent of an individual teachers’ engagement and development but rather a result of a kind of the above-mentioned spill-over effect, where a high performing and self-confident group is favoring an individuals’ thinking and acting. Comparably high levels of self-efficacy of some teachers might then merely originate from the fact that he or she is part of a successful and high performing team – without engaging themselves extra-ordinarily in a collaborative fashion (Ronfeldt et al., 2015).

Alternative suggestions, however, are needed to explain the negative age effect with respect to two subscales of teacher efficacy for the Swiss sample that is not accompanied by an effect with regard to years of teaching experience. Certainly, older teachers in Switzerland seem to be somewhat more reluctant or even skeptical with regard to their inclusion-related teaching performance or problem-solving capacity as a group of teachers. The fact that neither age nor job experience turns out to be a significant predictor of collaborative self-efficacy in the two other country samples does not point to a factor of general relevance. In sum, the monitoring of socio-demographic factors may well indicate strengths and weaknesses of certain school systems and should therefore be continued, especially with regard to success factors such as the collective teacher efficacy for inclusive educational practices.

## 6 Limitations

For each participating country occasional samples rather than representative samples where obtained, limiting the external validity of all results with regard to the respective school systems and the teachers therein. The invitations to partake in our study was freely distributed among teachers in Canada and Switzerland. Therefore, it is not known for these two countries in how far the participating teachers belonged to the same school or not. In Germany, however, the whole teaching staff of various schools from just one federal state (“Bundesland”) were invited to participate and it was possible to link each teachers’ response to his or her respective school, without interfering with the norm of anonymity. To a certain degree, therefore, the set-up of the three country-specific samples varies.

The factorial structure obtained only applies thus far to data from Canada, Germany, and Switzerland. Additional samples from Greece and Italy, however, did withstand configural and metric but not scalar invariance. It will be interesting to see if in the course of future studies the factorial structure presented in this paper will be reproduced or not and to learn more about the sample characteristics, study design details or the setup of the school systems that might explain the respective differences.

While our study accounts for institutional frameworks, the varying cultural interpretations of inclusivity – from Canada’s equity-centric model to Switzerland’s pragmatic integration – may influence teacher self-efficacy perceptions.

## 7 Conclusion

Using questionnaire data from teachers in Canada, Germany and Switzerland, and employing a multiple indicator multiple causes model (MIMIC) within an exploratory structural equation modelling (ESEM) framework, the tripartite structure of the TEIP scale measuring teachers’ individual efficacy beliefs to implement inclusive practices was confirmed with regard to a newly developed scale determining teachers’ collective efficacy beliefs to implement inclusive practices (TEIP-C). The present findings support the applicability of the scale originally developed and characterized by Sharma et al. (2024) for statistically sound international comparisons of school quality indicators, specifically teachers’ collective self-efficacy regarding *Inclusive Instructions*, *Managing Behavior* and *Collaboration* with parents and other school personnel. Due to the validation presented in this paper it now appears possible for researchers to freely combine either of the six subscales focusing on individual teachers’ efficacy or teachers’ collective efficacy with regard to inclusive education, depending on, e.g., research questions, questionnaire scope or length.

While the country samples did not differ with respect to *Inclusive Instructions*, significant differences in favor of Canadian teachers became apparent for *Collaboration* (Switzerland and Germany) as well as *Managing Behavior* (Germany). In total, the results underline the comparably high standards of inclusive teaching in Canada. Additional differences on the basis of the two subscales just mentioned pointed to somewhat lower ratings of collective teacher efficacy with respect to inclusive education by female teachers in Canada and Germany and older teachers in Switzerland.

## Data Availability

The raw data supporting the conclusions of this article will be made available by the authors, without undue reservation.
